# A landscape analysis of health technology assessment capacity in the Association of South-East Asian Nations region

**DOI:** 10.1186/s12961-020-00647-0

**Published:** 2021-02-11

**Authors:** Manushi Sharma, Yot Teerawattananon, Saudamini Vishwanath Dabak, Wanrudee Isaranuwatchai, Fiona Pearce, Songyot Pilasant, Junainah Sabirin, Mayfong Mayxay, Melissa Guerrero, Nguyen Khanh Phuong, Sudigdo Sastroasmoro, Thant Sin Htoo

**Affiliations:** 1 Health Intervention Technology Assessment Program (HITAP), Ministry of Public Health, 6th Floor, 6th Building, Tiwanon Road, Nonthaburi, 11000 Thailand; 2 Saw Swee Hock School of Public Health (SSHSPH), National University of Singapore (NUS), Singapore, Singapore; 3 Agency for Care Effectiveness (ACE), Ministry of Health, Singapore, Singapore; 4 Health Technology Assessment Section, Ministry of Health Malaysia (MaHTAS), Putrajaya, Malaysia; 5 Lao-Oxford-Mahosot Hospital-Wellcome Trust Research Unit (LOMWRU), Mahosot Hospital, Vientiane, Lao PDR; 6 Institute of Research and Education Development (IRED), University of Health Sciences, Ministry of Health, Vientiane, Lao PDR; 7 Health Technology Assessment Unit (HTAU), Manila, The Philippines; 8 Health Strategy and Policy Institute (HSPI), Hanoi, Vietnam; 9 Indonesian Health Technology Assessment Committee (InaHTAC), Jakarta, Indonesia; 10 Ministry of Health and Sports, Naypyidaw, Myanmar

**Keywords:** Health technology assessment, Survey, Health policy, Implementation research

## Abstract

**Background:**

Progress towards achieving Universal Health Coverage and institutionalizing healthcare priority setting through health technology assessment (HTA) in the Association of South-East Asian Nations (ASEAN) region varies considerably across countries because of differences in healthcare expenditure, political support, access to health information and technology infrastructure. To explore the status and capacity of HTA in the region, the ASEAN Secretariat requested for member countries to be surveyed to identify existing gaps and to propose solutions to help countries develop and streamline their priority-setting processes for improved healthcare decision-making.

**Methods:**

A mixed survey questionnaire with open- and closed-ended questions relating to HTA governance, HTA infrastructure, supply and demand of HTA and global HTA networking opportunities in each country was administered electronically to representatives of HTA nodal agencies of all ASEAN members. In-person meetings or email correspondence were used to clarify or validate any unclear responses. Results were collated and presented quantitatively.

**Results:**

Responses from eight out of ten member countries were analysed. The results illustrate that countries in the ASEAN region are at different stages of HTA institutionalization. While Malaysia, Singapore and Thailand have well-established processes and methods for priority setting through HTA, other countries, such as Cambodia, Indonesia, Lao PDR, Myanmar, the Philippines and Vietnam, have begun to develop HTA systems in their countries by establishing nodal agencies or conducting ad-hoc activities.

**Discussion and conclusion:**

The study provides a general overview of the HTA landscape in ASEAN countries. Systematic efforts to mitigate the gaps between the demand and supply of HTA in each country are required while ensuring adequate participation from stakeholders so that decisions for resource allocation are made in a fair, legitimate and transparent manner and are relevant to each local context.

## Key messages

- The ASEAN region has shown a strong commitment to achieving universal health coverage (UHC). Countries have taken different approaches to healthcare priority setting for UHC and offer rich lessons for other countries.
- This study provides a systematic description of HTA capacity in the ASEAN region.
- The results show that the basic infrastructure for HTA has been established; however, efforts to bridge the gaps between the demand and supply sides of HTA are required.
- In countries with limited HTA institutionalization, formalizing a system for HTA will reinforce the efforts for achieving UHC and promote good value for money while ensuring the provision of suitable healthcare services for the population.

## Background

The Association of South-East Asian Nations (ASEAN) region is diverse in terms of geography, society, economic development, and health systems and outcomes. Progress towards Universal Health Coverage (UHC) and institutionalization of healthcare priority setting through Health Technology Assessment (HTA), a formal multidisciplinary process that uses explicit methods to determine the value of a health technology (vaccines, drugs, devices, gene therapies, health interventions) at different points in its lifecycle [1], is varied in the region and is dependent on several country-specific factors such as the proportion of public investment in health, political support, access to good quality health information and technology infrastructure [2]. With the rising cost of healthcare, HTA can be a useful tool to inform decision-making about UHC and promote an equitable, efficient and high-quality health system [1]. As such, Brunei Darussalam, Malaysia, Singapore and Thailand provide UHC for their citizens and have an established system for setting healthcare priorities, which involves HTA, transparently and legitimately [2–5]. Other countries, such as Indonesia, the Philippines and Vietnam, have begun to provide UHC; however, priority-setting mechanisms and HTA processes remain at a nascent stage [2–6]. Lastly, in Cambodia, Lao People's Democratic Republic (PDR) and Myanmar, priority setting is conducted on an ad-hoc basis and is not part of routine decision-making processes [2]. While healthcare systems in these countries have unique characteristics, a series of common themes emerge such as, but not limited to, expertise and awareness of HTA, health information systems, public health infrastructure, investment in healthcare and stakeholder involvement, which act as facilitators or barriers for using HTA to inform UHC decisions [7–11].

Historically, the use of HTA has proven to be beneficial in setting healthcare priorities as seen in various middle-income countries such as Thailand and Brazil [12, 13] and high-income countries such as Australia, United Kingdom and other countries in the European Union [14, 15]. Recognizing that efficient use of resources is a crucial factor for ensuring the sustainability of health systems and achieving UHC, the WHO, in its resolution for the South-East Asian region, urged member states to pursue the HTA agenda as a priority [16]. In ASEAN, the role of HTA to inform healthcare decision-making has seen considerable advancements recently. There are several knowledge exchange opportunities which may lead to the overall improvement of the health systems and outcomes. As such, the ASEAN Secretariat under Health Cluster 3, which is responsible for *ensuring access to an affordable package of goods and services *[17], requested the Health Intervention and Technology Assessment Program (HITAP), Ministry of Public Health, Thailand, to assess HTA capacity among ASEAN countries. The overarching aim of this initiative by the ASEAN Secretariat is to assist member countries in developing and streamlining their healthcare priority-setting processes to improve decision-making for UHC. The objective of this survey is to explore the status and capacity of priority setting through HTA in each country in the ASEAN region.

## Methods

### Survey design

A mixed survey questionnaire with open- and closed-ended questions was designed based on a review of existing literature, knowledge of HTA in the Asia-Pacific region and previous surveys of a similar nature [9, 18–20]. The questionnaire was divided into four sections to provide a comprehensive snapshot of the HTA landscape in ASEAN members, as listed below:

- HTA Governance: HTA varies from country to country (or even between regions and provinces within individual countries). This section aims to understand the current governance (establishment of policies or structures that mandate the use of evidence for health decision-making) in member countries and any gaps which hinder implementation and institutionalization of HTA.
- HTA Infrastructure: This section aims to understand the basic organizational structures and pathways that reinforce or weaken the use of HTA in local settings.
- Demand and Supply for HTA: This section aims to understand who the users (consumers who create demand) and producers (suppliers who cater to the demand) of HTA are.
- Networking in HTA: This section aims to understand the global networking activities in HTA that member countries participate in.

The questionnaire was made available only in the English language and was piloted with representatives in Singapore and Thailand. The content was amended based on the feedback received to ensure questions were clearly described and appropriate for the scope of the project.

### Survey sample

The ASEAN region comprises ten member countries, Brunei Darussalam, Cambodia, Indonesia, Lao PDR, Malaysia, Myanmar, Singapore, Thailand, the Philippines and Vietnam. The sample was selected purposively, that is, programme leaders or representatives of HTA nodal agencies of all ASEAN members. In countries where HTA nodal agencies were unavailable such as Lao PDR, Cambodia, Myanmar and Brunei Darussalam, representatives from relevant government departments and universities were requested to complete the survey. The total number of respondents for this survey (n) was eight, representing Indonesia, Lao PDR, Malaysia, Myanmar, the Philippines, Singapore, Thailand and Vietnam. There was no response from Brunei Darussalam. Responses from Cambodia were received but were not combined with responses from the other countries as the information provided was incomplete, given the health system is still developing and HTA processes have not been sufficiently established yet.

### Survey administration

The survey was administered online over a 2-month period (October – November 2019). Given the geographical spread, correspondence with the respondents was via email. In the case where answers were not clear or conflicted with the existing literature, the lead author conducted in-person discussions (Cambodia and Thailand) or followed up via email (Malaysia) to clarify specific information with the respondents.

### Survey analysis

Descriptive statistics were employed to present the results of the rest of the data collected quantitatively. Parts of the write-up were substantiated by a literature review.

## Results

Results were organized into three sections: (1) governance and infrastructure of HTA in ASEAN—this section explains the legislative requirements and basic guidelines that define the remit of HTA in ASEAN countries. It also shows the stakeholders that are involved in various stages of the HTA process; (2) producers and users of HTA in ASEAN—this section describes the nature of demand and supply of HTA in each country and the networking opportunities that countries participate in to strengthen HTA capacity; (3) limitation in the institutionalization of HTA in ASEAN—this section describes limitations of HTA governance, HTA infrastructure and translation of research into policy that act as barriers in the formalization of HTA processes and procedures.

### Governance and Infrastructure of HTA in the ASEAN

#### Legislative requirements for HTA

Among respondents, seven out of eight countries (Indonesia, Lao PDR, Malaysia, Myanmar, Singapore, Thailand and Vietnam) reported not having a formal legislative mandate from the government to use HTA to inform healthcare decision-making and allocation of resources for UHC. However, HTA can be used to inform decisions on certain types of health technologies, especially those which are high cost in these countries (Table 1). Six countries (Indonesia, Malaysia, Singapore, Thailand, the Philippines and Vietnam) also confirmed that they have HTA nodal agencies within government bodies such as their ministries of health**.** The government of Lao PDR is planning to establish the Unit for Health Evidence and Policy (UHEP) to function as the nodal agency for all HTA activities.

**Table 1:** Legislations for HTA

Country	Legislative mandate	Other legal provisions	HTA nodal agency
Indonesia	No	Yes	Indonesian Health Technology and Assessment Committee (InaHTAC)
Lao PDR	No	No	Unit for Health Evidence and Policy (UHEP) Will be established to serve as focal agency for all HTA activities
Malaysia	No	Yes	Malaysian Health Technology Assessment Section (MaHTAS)
Myanmar	No	No	
Singapore	No	Yes	Agency for Care Effectiveness (ACE)
Thailand	No	Yes	Health Intervention and Technology Assessment Program (HITAP)
The Philippines	Yes	No	The Department of Health’s Health Technology Assessment Unit (HTAU)
Vietnam	No	Yes	Health Strategy and Policy Institute (HSPI) A unit dedicated to HTA will be established soon

Legislative mandate—law or decree for use and implementation of HTA for healthcare decision-making

Other legal provisions—by-laws or amendments that mandate the use of HTA for certain category of drugs thereby allowing for the establishment of HTA bodies

In Thailand, HITAP, a semi-autonomous unit in the Ministry of Public Health, serves as the nodal agency for HTA to inform: (i) decisions related to high-cost drugs (E2) which fall under the National List of Essential Medicines; (ii) assessments of medical devices as mandated by the Medical Device Act B.E. 2551; (iii) updates to the benefits package that is provided under the Universal Coverage Scheme which is managed by the National Health Security Office [4, 12]. In Singapore, the Agency for Care Effectiveness (ACE) was established in August 2015 under the Ministry of Health as the national HTA agency to evaluate health technologies for subsidy consideration. However, although HTA processes are now embedded in decision-making, specific legislation has not been passed in Singapore mandating HTA to be used to inform health policy decisions [21]. Similarly, Malaysia has a policy document requiring HTA to be conducted before health technologies, especially expensive technologies, can be procured [3, 22]. In Indonesia, presidential regulation 12/2013 [23] mandated the use of HTA, and subsequently the Health Technology Assessment Committee (InaHTAC) was established in 2013. In other countries, HTA is still at a very nascent stage. Nevertheless, as health systems undergo restructuring to align with national health priorities, priority setting is at the forefront of all discussions. With the passage of the UHC Act in the Philippines in 2019 [6], the country has solidified its efforts to institutionalize HTA by setting up a Health Technology Assessment Unit (HTAU). The development of the HTA process and methods guidelines by the HTAU under the supervision of the HTA council is ongoing. Vietnam, in 2013, assigned the Health Strategy and Policy Institute (HSPI) to be the focal point for the development of HTA. Although the use of HTA in Vietnam is still at an early stage, it has become necessary to support policymakers in making difficult healthcare resource decisions, and a unit dedicated to HTA will soon be established [24]. In the remaining countries, that is, Myanmar and Lao PDR, HTA research is only conducted occasionally as these countries are in the early stages of establishing information systems and developing HTA capabilities. Furthermore, these countries are heavily reliant on donor aid, and healthcare resource allocations reflect these priorities.

While six out of eight countries (Indonesia, Malaysia, Singapore, Thailand, the Philippines, Vietnam) reported having standard guidelines defining their local processes and methods for HTA, the role of HTA in decision-making and the use of cost-effectiveness thresholds or other mechanisms to determine what constitutes an appropriate use of healthcare resources in each local context are still under development. Most of the respondent countries use a fixed cost-effectiveness threshold which has either been set by their country or is in line with the arbitrary thresholds proposed by WHO [25] (Table 2).

**Table 2:** Cost-effectiveness thresholds in the ASEAN region

Countries	Country-specific cost-effectiveness threshold?	Practical cost-effectiveness threshold used
Indonesia	No	3 times per capita GDP
Lao PDR	No	Not yet implemented
Malaysia	Yes	Less than or equal to 1 GDP per capita/QALY gain
Myanmar	No	1–3 GDP per capita (previous WHO recommendation)
Singapore	No	None—cost-effectiveness is one of several factors considered by decision-makers when making funding decisions. Decision-makers consider the upper and lower limits of the incremental cost-effectiveness ratio (ICER) range, in addition to the base-case point estimate when determining whether a technology represents good value for money
Thailand	Yes	US$ 5250
The Philippines	No	There is no explicit threshold for cost-effectiveness as this decision criterion is one of the various factors considered by the HTAC in making funding recommendations. Other factors include burden of disease, clinical effectiveness, affordability, equity, social acceptability, feasibility and health system implications. Cost-effectiveness is weighed together with the other relevant criteria
Vietnam	No	1–3 GDP per capita (previous WHO recommendation)

#### Considerations for conducting HTA

**Fig. 1:**
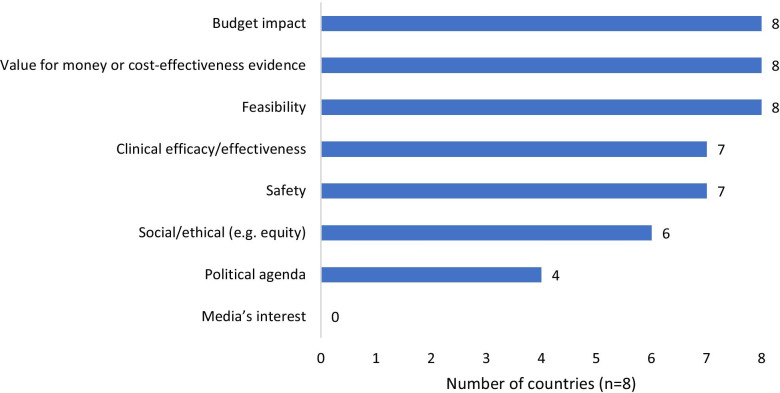
Considerations for conducting HTA

HTA is a formal, systematic process designed to synthesize and evaluate the existing evidence for prioritization of medical technologies (vaccines, drugs, devices, gene therapies, health interventions). Depending on the perspective, the research process includes a multi-faceted assessment of clinical and safety outcomes, economic consequences, social and ethical factors and the feasibility of implementation of recommendations, among others. As shown in Fig. 1 all respondents said that cost-effectiveness and budget impact analyses of select technologies, in addition to feasibility of implementing recommendations, are important considerations for conducting an HTA. Additional important considerations include expected clinical outcomes and safety. Social and ethical considerations, political agenda and media interest ranked as the lowest considerations for conducting an HTA. Compared to other ASEAN countries, Myanmar does not consider safety or clinical efficacy/effectiveness. Myanmar and Vietnam do not take social and ethical considerations into account. Lastly, Lao PDR, Myanmar, Singapore and Vietnam are the only countries that consider the political agenda when prioritizing technologies for evaluation.

#### Applications of HTA

Traditionally, HTA is used for the purpose of *introducing* technologies in a health system. However, health technologies are ‘moving targets’ for assessment [26]. With the advancement of medical science and innovation of medical technologies, HTA should be an iterative process and can also be useful when applied for *reassessment* of existing technologies.

All respondents said that HTA is predominantly used for the introduction of new medical technologies (Table 3). Indonesia has a dedicated unit that conducts all vaccine-related assessments (ITAGI), and the HTA Committee (InaHTAC) assesses all other interventions. Myanmar uses HTA to inform decisions about the funding of vaccines, health screening programmes and public health interventions, but not drugs. In Lao PDR, HTA is currently required only for vaccines. Singapore’s agency (ACE) does not currently conduct HTA for health screening programmes and public health interventions, beyond vaccines. These assessments are conducted by individual healthcare institutions or academic centers to address specific research questions.

**Table 3:** Applications of HTA

Country	Drugs		Vaccines		Devices		Health screening programs		Public health interventions	
	Introducing	Reassessment	Introducing	Reassessment	Introducing	Reassessment	Introducing	Reassessment	Introducing	Reassessment
Indonesia	✓	✓	NA*	NA*	✓	✓	✓	✓	✓	✓
Lao PDR	NA	NA	✓	NA	NA	NA	NA	NA	NA	NA
Malaysia	✓	✓	✓	✓	✓	✓	✓	✓	✓	✓
Myanmar	NA	NA	✓	✓	NA	NA	✓	✓	✓	✓
Singapore	✓	✓	✓	✓	✓	✓	NA	NA	NA	NA
Thailand	✓	NA	✓	NA	✓	NA	✓	NA	✓	NA
The Philippines	✓	✓	✓	✓	✓	✓	✓	✓	✓	✓
Vietnam	✓	✓	✓	NA	✓	✓	NA	NA	NA	NA

“Introducing” refers to conducting HTA to inform decisions about new medical technologies

“Reassessment” refers to conducting HTA to reassess the existing medical technologies

*NA*—not available

*The Indonesian Technical Advisory Group on Immunization (ITAGI) is the nodal agency for all vaccine-related matters

#### Stakeholders involved in each step of the HTA process

Apart from HTA technical capacity, institutional arrangements are critical in ensuring that a credible and transparent assessment process can be established to translate evidence into policy in each local context [20]. A key success factor in achieving this outcome is to ensure that all stakeholders have a good understanding of HTA and have sufficient support to allow them to meaningfully contribute to HTAs [9, 10].

**Fig. 2:**
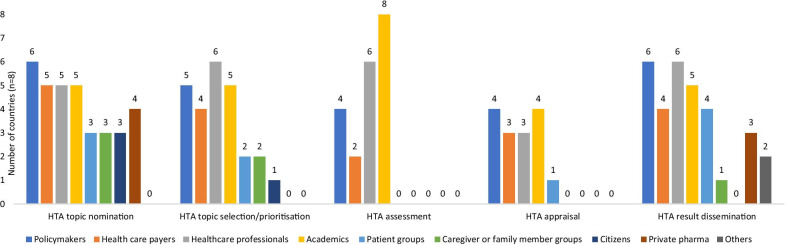
Stakeholders in the HTA process

This study reveals that stakeholder involvement varies among countries for each aspect of the HTA process (topic nomination, topic prioritization, HTA assessment, HTA appraisal and result dissemination). Respondents confirmed that policymakers are involved in all five aspects of the HTA process followed closely by the health care payers. Technical capacity to perform HTA is concentrated among academics and researchers, who are heavily involved in step 3 (HTA assessment) (Fig. 2), as indicated by all respondent countries. The public and caregiver groups are the least involved in the entire HTA process across all countries. Only Indonesia, Myanmar, Singapore and Thailand allow private pharmaceutical firms to be involved in topic nomination and to provide evidence to inform HTAs as part of their process. In Malaysia, MaHTAS, accepts HTA requests from clinicians, programme heads and the Pharmaceutical Services Division (PSD). The PSD accepts submissions from pharmaceutical firms while preparing a dossier to list technologies in the national formulary. Overall, stakeholder involvement can be improved across all aspects of the HTA process to promote transparency in assessment and decision-making processes and ensure final decisions are relevant and feasible to implement. Concurrently, an accurate record must be maintained of any conflict of interest for the stakeholders involved.

### Producers and users of HTA

#### Users and funders of HTA

The majority of the respondent countries, namely, Indonesia, Lao PDR, Malaysia, Singapore, Thailand, the Philippines and Vietnam, said that government authorities such as benefits package committees, essential medicines committees and food and drug administrations are the main users of HTA in their local contexts. As reported by Indonesia, Lao PDR, the Philippines and Vietnam, the most popular international healthcare agencies, which are also users and funders of HTAs, are Rockefeller Foundation, Department for International Development (DFID), United States Agency for International Development (USAID), WHO and the United Nations Children’s Fund (UNICEF). Public health providers or healthcare units that administer the national UHC programme are also users and/or funders of HTA work in Indonesia, the Philippines, Singapore, Thailand and Vietnam. Interestingly in Lao PDR, multinational pharmaceutical firms are occasionally cited as the users and funders of HTA.

*Type of information sought by the users* Respondents reported that the majority of users in all countries, except Myanmar, require information about the cost-effectiveness and budget impact of the intervention to inform decisions about health policy and planning. For evidence to inform policy, it is crucial that the users of HTA, that is, high-level managers, decision-makers, etc., are equipped to accurately interpret the findings of an HTA study to inform their decisions. The capacity of the users also influences the translation of research into policy or the demand for HTA. In the surveyed group of countries, five out of eight countries, namely Indonesia, Lao PDR, the Philippines, Singapore and Thailand, said they have periodic capacity-building activities specifically for their users of HTA.

#### Producers of HTA

Among the surveyed countries, respondents from six out of eight countries, that is, Indonesia, Lao PDR, Malaysia, Singapore, Thailand and Vietnam, said that the main producers of HTA are HTA nodal agencies, local universities or the academics, followed by healthcare institutes. This finding is also corroborated by the results shown in Fig. 2 wherein academics are the stakeholders conducting HTAs in most countries. In Lao PDR and Vietnam, private pharmaceutical companies also produce HTA studies. In Malaysia, private pharmaceutical companies can submit their requests/dossiers to the PSD, and in case of ambiguity, the PSD will approach MaHTAS for an in-depth assessment. In other countries, private pharmaceutical companies can provide evidence to inform HTAs conducted by nodal agencies. None of the countries allow pharmaceutical companies to be involved in recommending the health technology for reimbursement.

##### HTA nodal agencies in ASEAN countries

Table 4 is a summary of the HTA nodal agencies that have been established in ASEAN countries.

**Table 4:** HTA units in ASEAN countries

Country	HTA agency	Year of establishment		Governance (autonomous/semi-autonomous/government)	No. of professionals	No. of HTAs conducted	Types of technologies assessed
Indonesia	Indonesian Health Technology Assessment Committee (InaHTAC)	2014	Government		10	11	Drugs, devices, health interventions
Malaysia	Malaysian Health Technology Assessment Section (MaHTAS)	1995	Government		33	438*	Drugs, medical devices, programmes, procedures
Singapore	Agency for Care Effectiveness (ACE)	2015	Government		74 (only 50 are involved with HTA)	120**	Drugs, vaccines, gene therapies, medical technologies (devices and medical services)
Thailand	Health Intervention and Technology Assessment Program (HITAP)	2007	Semi-autonomous		68	228	Drugs, vaccines, devices, health interventions
The Philippines	Health Technology Assessment Unit (HTAU)	2019	Semi-autonomous		45	15	Drugs, vaccine, devices, interventions, traditional medicine
Vietnam	Health Strategy and Policy Institute (HSPI)	2013	Government		A unit explicitly for HTA is expected to be established. The specifics are to be decided		Drugs, devices, health interventions

Table as of March 2020

*Including rapid/mini HTAs

**Excluding rapid/mini HTAs

#### Networking

The HTAsiaLink network was cited by respondents from six out of eight countries as the most popular international HTA networking activity to keep members abreast of developments in the HTA field within the region and globally.

### Limitations in the institutionalization of HTA

This section describes the limitations that are common to the ASEAN countries which can impede the institutionalization of HTA in each local context.

#### Limitations in HTA governance

Respondents from all countries considered that not having adequate funding can have a major impact on the supply of HTA, followed closely by lack of political support and lack of understanding of HTA processes by users/decision-makers. Half of the respondent countries, that is, Indonesia, Lao PDR, the Philippines and Vietnam, said that the risk of private sector interference is also a concern that can affect HTA outputs and robust decision-making. Indonesia, Myanmar and Vietnam cited lack of transparency in the decision-making process and ill-managed risk of conflict as issues that hamper the progress of HTA institutionalization and governance in their local settings.

#### Limitations in HTA infrastructure

HTA is a data-intensive exercise, and without basic information systems in place on a national level, the exercise to obtain adequate data to inform HTAs is cumbersome and unreliable. All the respondent countries reported that access to local data is a big obstacle for robust HTA research. Furthermore, despite HTA activities gaining traction and acceptance by high-level policymakers in recent years and efforts being made to systemize HTA for priority setting, all countries except Singapore considered there is a lack of local technical capacity to conduct HTA in their settings. Half of the respondents, that is, Indonesia, Lao PDR, Myanmar and Vietnam, also reported that an absence of country focal points or champions for HTA activities, a lack of standard procedures for performing HTA and a lack of expertise in implementing HTA decisions are other factors that negatively impact the development of the HTA infrastructure in their countries.

#### Limitations in the translation of research to policy

**Fig. 3:**
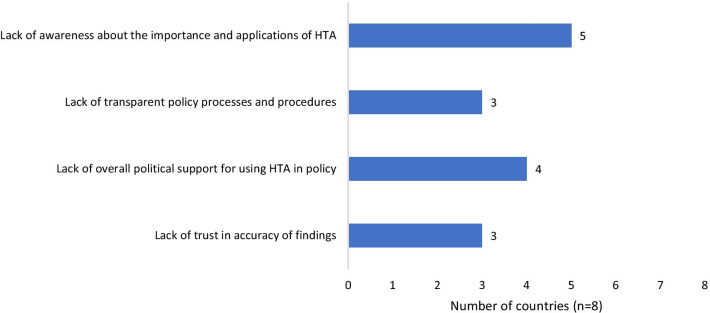
Limitations in translation of research into policy

All countries, except the Philippines, reported a lack of awareness about the importance and application of HTA in their local settings, as the main factor hindering the mobilization of research into policy. This lack of awareness also contributes to a lack of overall political support for the uptake of HTA in health policy as reported by four countries (Indonesia, Lao PDR, Malaysia and Vietnam). Lastly, the lack of transparent policies and procedures to incorporate HTA into decision-making also contributes to the gap in the translation of research to policy in three countries (Indonesia, Lao PDR and Vietnam) as seen in Fig. 3.

## Discussion

This survey provides a systematic description of HTA capacity in ASEAN countries, intending to inform areas which can be strengthened through further research and collaborations. Results show that most countries have established basic infrastructure for HTA although efforts to bridge the gaps between demand and supply of HTA are required to ensure that work conducted is useful to inform policy for achieving and sustaining UHC.

### Key recommendations

- Explore opportunities to institutionalize the use of HTA for healthcare decision-making, including legislation, as appropriate to the context.
- Enhance capacity of both users (demand side) and producers (supply side) of HTA to foster a culture of using contextual evidence in decision-making.
- Critically appraise international guidelines on cost-effectiveness thresholds before implementation to ensure relevance to the local context.
- Ensure equal representation from all stakeholders, that is, policy makers, technical (economists, academics, doctors and public health practitioners) and non-technical groups (civil society, patient organizations) to improve the legitimacy of the process and the relevance of the decisions.
- Diversify application of HTA to reassessment of existing technologies and policies as well as other areas.
- Utilize existing networks such as iDSI and HTAsiaLink to undertake activities to build HTA capacity for ASEAN members.

The increase in demand for healthcare priority setting through HTA is demonstrated by the fact that most ASEAN countries have an HTA nodal agency, despite not having explicit legislation in place. However, the majority of the country representatives highlighted that inadequate political support prevents institutionalization of HTA and an explicit legislation could help with acceptance and adoption of HTA and potentially generate greater demand for robust HTAs.

Results show that all countries acknowledge the importance of *value for money* of health technologies for conducting HTAs and cited it as one of the main considerations, in contrast to being on the political agenda, which is considered least important. While HTA agencies and studies must be free from political interference, ensuring evaluations are conducted in line with national priorities, with support from key political stakeholders is crucial to drive adoption of HTA recommendations and ensure successful translation of research into policy. As adduced by the ‘triangle that moves the mountain’ approach, *political involvement* is an essential part of the triad along with *the creation of relevant knowledge* and *social movement *[27]*.* Translation deficit can be remedied through targeted political advocacy to drive policy development and implementation alongside reinforced efforts to increase stakeholder involvement in the HTA process [27–30]. A Cochrane review has also shown that identifying ‘champions’ or ‘opinion leaders’ is needed to bridge the research-to-policy gap [30].

Positively, results confirm that most countries have defined the remit and set standards for HTA in their local settings. However, several countries are using international recommendations (e.g., from WHO) when ascertaining the cost-effectiveness of technologies in their local settings, without checking for transferability or feasibility. While international guidelines are a good starting point, they have been criticized for lacking nuance and context; therefore, meticulous scrutiny is recommended before implementation [31, 32]. The WHO threshold does not reflect the opportunity costs imposed on health care systems and is widely debated by health economists as being largely out of date [33]. As evidenced by the literature, allocation decisions based on WHO thresholds are likely to recommend interventions that can lead to reductions in population health causing more long-term harm [31, 33–36]. Thus, ASEAN countries are advised to apply these thresholds and all international recommendations with caution.

ASEAN members have been applying HTA primarily to inform decisions about the introduction of new health technologies. Given that health systems and HTA processes in ASEAN countries are still largely evolving, this finding is not surprising. However, with increasing innovation and a wide variety of medical technologies available, applying HTA for reassessment of the existing benefits packages and essential medicine lists would ensure smart decisions on investments or could assess the impact of the existing technologies on patient outcomes. Reassessment allows decision-makers to optimize the use of healthcare technologies to achieve the greatest clinical benefit as has been evidenced from global examples, highlighting their relevance for LMICs within ASEAN, which have limited healthcare resources [37]. As corroborated by examples from the United Kingdom, Australia, Brazil and the Republic of Korea [37–39], if applied correctly, reassessment can be embedded into the regular HTA process and applied as a tool to avoid wasteful buys. However, usage of reassessment would require stakeholder input, especially from clinicians, patient organizations and the public to establish a consensus on matters of ethics to avoid conflict with social values and alleviate inadvertent effects [37].

The survey reveals that most countries have established a formal process for HTA, but stakeholder involvement can be improved as public and patient perspectives are underrepresented. Setting clear priorities in health is not a straightforward exercise, and HTA production is part of a larger societal process [9, 19, 20, 26]. As such, the utilization of HTA evidence depends on successful interaction with different kinds of stakeholders to ensure that the final output is relevant.

Stakeholders must be provided with adequate support and training to enable them to interpret and use the findings of HTA and to meaningfully contribute to HTA processes. Furthermore, for HTA research to legitimately inform policy decisions, capacity building needs to have a holistic approach, and all stakeholders should understand and appreciate the importance of priority setting. From the survey responses, it can be inferred that capacity for HTA among all stakeholders is moderate to low in most ASEAN countries apart from Malaysia, Singapore and Thailand; therefore, more local initiatives will be required to make HTA an inclusive and transparent process. The survey shows that countries are undertaking capacity-building initiatives for the users or demand-side of HTA and active efforts are needed to mitigate the limitations that have been voiced by members, such as lack of funding, political support and awareness about the importance and applications of HTA.

As highlighted in the survey, on the supply side of HTA, most ASEAN countries lack sufficient HTA infrastructure, such as access to local data on costs, clinical information and health outcomes, for informing clinical and economic evaluations. Furthermore, local technical expertise to perform HTA is currently concentrated among researchers and academics in universities, or in nodal HTA agencies, which is insufficient to cater to the rising demand for priority setting in ASEAN countries. Training for more working-level staff and targeted technical support for HTA capacity building, for example, the International Decision Support Initiative (iDSI), can provide the impetus that is required. However, in the long term, success and sustainability of any HTA initiative will be decided by the level of local involvement and ownership. Instituting specialized programmes such as post-graduate courses (masters, PhDs) or vocational training on HTA in national universities with guaranteed employment opportunities in the public health system will address the paucity of skilled professionals as well as ensure a locally led HTA agenda. The Thai capacity-building initiative in HTA is an excellent example [20], and the Philippines is set to pursue a similar approach. It is also important that suppliers of evidence can understand the immediate demand of health systems and policymakers to link their research with policy, which also corresponds to the research-to-policy-gap issue raised earlier [9, 20]. Revising human resource policies and employee benefit packages will ensure retention of experienced technical staff in the public health system.

Regional networks such as EUNetHTA and the Pan American Health Organization (PAHO) have proven to be beneficial for ASEAN countries which have been able to learn from their experiences and address similar contextual challenges [14, 40]. Networks are beneficial in linking the research community to decision-makers and professional regulators, and a similar network for ASEAN is advised. Considering that respondents acknowledge HTAsiaLink as the most popular network among ASEAN countries, a working group dedicated to ASEAN countries could be a starting point where countries with nascent HTA systems can learn from those with more mature HTA systems.

There are certain limitations to the study. Results of the survey have been synthesized based on responses from one key individual from each country, who was identified as being best placed to provide a comprehensive snapshot of the capacity of public sector HTA initiatives in their respective countries. Parts of the narrative have been substantiated by literature reviews, and requests for official documentation from the respondents were made to validate their responses. It should be noted that a representative from Cambodia also responded to the survey; however, given the nascent stage of their HTA processes (and the health system overall), the information provided was found to be inadequate for the purposes of this study. Therefore, responses from Cambodia were noted but not combined with those from other countries. Future research may be conducted to overcome these limitations and may involve conducting in-depth country-specific analyses that capture all stakeholders involved in the production and use of HTA to generate contextualized evidence for informed policy making.

## Conclusion

Overall, this study provides a useful overview of the current HTA landscape in most ASEAN countries. As countries strive to deliver UHC, institutionalization of HTA becomes an indispensable task. ASEAN members are actively paving the way forward for improving the process of evidence-informed priority setting by bringing together academics, economic experts, physicians, allied health professionals and policy makers, along with international experts. Systematic efforts to mitigate the gaps between the demand and supply side of HTA in each country are required to ensure that decisions for resource allocation are made in a fair, legitimate and transparent manner and are relevant to each local context.

## Supplementary information

**Additional file 1:** Assessment of HTA capacity in ASEAN.

## Data Availability

Have been appended as Additional file 1: Questionnarie.

## Abbreviations

Laos PDR: Lao People’s Democratic Republic
HTA: Health technology assessment
ASEAN: Association of South-East Asian Nations
UHC: Universal health coverage
WHO: World Health Organization
HITAP: Health Intervention and Technology Assessment Program
GDP: Gross domestic product
QALY: Quality adjusted life-years
USD: United States dollar
ICER: Incremental cost-effectiveness ratio
UNICEF: United Nations International Children’s Fund
LMIC: Low- and middle-income countries

## References

[1] O’Rourke B, Oortwijn W, Schuller T (2020). The new definition of health technology assessment: A milestone in international collaboration. Int J Technol Assess Health Care.

[2] Van Minh H, Pocock NS, Chaiyakunapruk N (2014). Progress toward universal health coverage in ASEAN. Glob Health Action.

[3] Roza S, Junainah S, Izzuna MMG (2019). Health Technology Assessment in Malaysia: past, present, and future. Int J Technol Assess Health Care.

[4] World Health Organization. Regional Office for the Western Pacific. The Kingdom of Thailand health system review. Manila: WHO Regional Office for the Western Pacific. 2015. https://apps.who.int/iris/handle/10665/208216.

[5] Progressive realization towards Universal Health Coverage: ASEAN Member States. Bangkok, Thailand: 2019. https://www.aseanstats.org/infographics/asean-statistical. Accessed 3 Apr 2020.

[6] Universal Health Care Act—Republic Act No. 11223. 2018. https://www.officialgazette.gov.ph/downloads/2019/02feb/20190220-RA-11223-RRD.pdf. Accessed 15 Mar 2020.

[7] Myint C-Y, Pavlova M, Thein K-N-N (2019). A systematic review of the health-financing mechanisms in the Association of Southeast Asian Nations countries and the People’s Republic of China: lessons for the move towards universal health coverage. PLoS ONE.

[8] Sharma M, Teerawattananon Y, Luz A (2020). Institutionalizing Evidence-informed priority setting for universal health coverage: lessons from Indonesia. Inq J Heal Care Organ Provision Financ.

[9] Teerawattananon Y, Rattanavipapong W, Lin LW (2020). Landscape analysis of health technology assessment (HTA): systems and practices in Asia. Int J Technol Assess Health Care.

[10] Teerawattananon Y, Teo YY, Dabak S (2020). Tackling the 3 big challenges confronting health technology assessment development in Asia: a commentary. Value Heal Reg Issues.

[11] MacQuilkan K, Baker P, Downey L (2018). Strengthening health technology assessment systems in the global south: a comparative analysis of the HTA journeys of China, India and South Africa. Glob Health Action.

[12] Leelahavarong P, Doungthipsirikul S, Kumluang S, Poonchai A, Kittiratchakool N, Chinnacom DSN, Tantivess S (2019). Health Technology Assessment in Thailand: institutionalization and contribution to healthcare decision making: review of literature. Int J Technol Assess Health Care.

[13] Oortwijn W, Broos P, Vondeling H (2013). Mapping of Health Technology assessment in selected countries. Int J Technol Assess Health Care.

[14] European Network of Health Technology Assessment—EUnetHTA. 2020. https://www.eunethta.eu/. Accessed 10 Sept 2019.

[15] Tantivess S, Chalkidou K, Tritasavit N (2017). Health Technology Assessment capacity development in low- and middle-income countries: Experiences from the international units of HITAP and NICE. F1000Research.

[16] [SEA/RC66/R4] Resolution of the World Health Organization Regional Committee for South East Asia. Health Intervention and Technology Assessment in Support of Universal Health Coverage. 2007. https://www.who.int/medical_devices/assessment/resolutionsearo_searc66r4.pdf?ua=1. Accessed 17 June 2020.

[17] Association of Southeast Asian Nations. A. Health Cluster 3: Strengthening Health System and Access to Care. https://asean.org/wp-content/uploads/2017/02/Agd-8.3_3.-ASEAN-Health-Cluster-3-Work-Programme_Endorsed-SOMHD.pdf. Accessed 31 Mar 2020.

[18] Health Intervention and Technology Assessment Program. Questionnaire—selecting common challenges in institutionalisation of HTA. 2019;1–10.

[19] World Health Organization. 2015 Global Survey on Health Technology Assessment by National Authorities—main findings. Geneva: Switzerland: 2015. https://www.who.int. Accessed 25 Mar 2019.

[20] Chootipongchaivat S, Tritasavit N, Luz A, et al. Conducive Factors to the development of Health Technology Assessment (HTA) in Asia. https://www.idsihealth.org/wp-content/uploads/2016/02/CONDUCIVE-FACTORS-TO-THE-DEVELOPMENT_resize.pdf. Accessed 16 Oct 2018.

[21] Pearce F, Lin L, Teo E (2019). Health Technology Assessment and Its Use in Drug Policies: Singapore. Value Heal Reg Issues.

[22] Health Technology Assessment Manual - Malaysia. 2015. https://apps.who.int/medicinedocs/documents/s22255en/s22255en.pdf (accessed 15 Mar 2020).

[23] Translation—Presidential Regulation no. 12/2013. 2013. https://www.social-protection.org/gimi/gess/RessourceDownload.action?ressource.ressourceId=40498.

[24] Kieu T (2017). Health Technology Assessment and Its Application in Vietnam. Value Health.

[25] Hutubessy R, Chisholm D, Edejer T (2003). Generalized cost-effectiveness analysis for national-level priority-setting in the health sector. Cost Eff Resour Alloc.

[26] Goodman CS. HTA 101: Introduction to Health Technology Assessment. 2014. https://www.nlm.nih.gov/nichsr/hta101/HTA_101_FINAL_02-02-15.pdf. Accessed 18 Feb 2020.

[27] Wasi P. ‘Triangle That Moves The Mountain’ and Health Systems Reform Movement in Thailand. 2000.

[28] FHI 360. Eight strategies for research to practice. 2012. https://www.fhi360.org/. Accessed 13 Mar 2020.

[29] Martin K, Mullan Z, Horton R (2019). Comment Overcoming the research to policy gap. Lancet Glob Health.

[30] Flodgren G, Parmelli E, Doumit G (2011). Local opinion leaders: effects on professional practice and health care outcomes. Cochrane Database Syst Rev.

[31] Turk F (2010). Data generalizability, data transferability, and the political economy of pharmacoeconomic guidelines. Value Health.

[32] Barbieri M, Drummond M, Rutten F (2010). What do international pharmacoeconomic guidelines say about economic data transferability?. Value Health.

[33] Woods B, Revill P, Sculpher M (2016). Country-level cost-effectiveness thresholds: initial estimates and the need for further research. Value Heal.

[34] Bertram MY, Lauer JA, De Joncheere K (2016). Policy & practice Cost-effectiveness thresholds: pros and cons. Bull World Heal Organ.

[35] Leech AA, Kim DD, Cohen JT (2018). Use and misuse of cost-effectiveness analysis thresholds in low-and middle-income countries: trends in cost-per-DALY studies. Value Health.

[36] Robinson LA, Hammitt JK, Chang AY (2017). Understanding and improving the one and three times GDP per capita cost-effectiveness thresholds. Health Policy Plan.

[37] MacKean G, Noseworthy T, Elshaug GA (2013). Health Technology Reassessment: the art of the possible. Int J Technol Assess Health Care.

[38] Seo HJ, Park JJ, Lee SH (2016). A systematic review on current status of health technology reassessment: Insights for South Korea. Health Res Policy Syst.

[39] Pereira VC, Barreto JOM, da Neves FAR (2019). Health technology reassessment in the Brazilian public health system: Analysis of the current status. PLoS ONE.

[40] Health Technology Assessment (HTA). 2020. https://www.paho.org/hq/index.php%3Foption%3Dcom_content%26view%3Darticle%26id%3D9229:2013-tecnologias-sanitarias%26Itemid%3D41687%26lang%3Den. Accessed 13 Mar 2020.

