# Supplementary material for: A landscape analysis of health technology assessment capacity in the Association of South-East Asian Nations region
Source: Health Res Policy Syst. 2021 Feb 11;19:19. doi: 10.1186/s12961-020-00647-0 (PMC7879649; doi:10.1186/s12961-020-00647-0)
Supplement: Supplementary file 1 — Additional file 1: Assessment of HTA capacity in ASEAN. [file 12961_2020_647_MOESM1_ESM.pdf]

Assessment of HTA capacity in ASEAN – Questionnaire

Health Technology Assessment is a multidisciplinary process that summarises information about medical, social, economic and ethical issues related to the use of health technology in a systematic, transparent, unbiased, robust manner.[1] In the ASEAN region, HTA has increasingly gained popularity as more countries strive to institutionalise evidence-based priority setting and achieve universal health coverage. For example, HTA is a tool for evaluating medicines, vaccines, devices, and public health interventions before including or excluding these technologies in the publicly subsidised programs.

Under the *Health Cluster Strategies* of ASEAN, Health Intervention Technology Assessment Program (HITAP), Thailand is assigned to conduct a survey to assess the current situation of technical capacity in HTA in ASEAN countries. The results will inform regional and national strategies of the ASEAN and its members to address the capacity gap. Also, HITAP will develop an academic report for publication in international journals and will include all respondents as co-authors. Please communicate to us if you would like to maintain the confidentiality of certain answers.

The survey has **five** sections:

I. HTA Governance
II. HTA Infrastructure
III. Demand for HTA
IV. Supply for HTA
V. Networking in HTA

Thank you for agreeing to take part in this important survey. It should take up to **45 mins – 60 mins**. For any clarifications, please write to manushi.s@hitap.net

[1] European Network of Health Technology Assessment - EUnetHTA. https://www.eunethta.eu/

Please state your

Name: ____

Position: ____

Organisation:____

Country: ____

I. **HTA Governance**

Health Technology Assessment varies from country-to-country (or even region and province). This section aims to understand the current governance (establishment of policies or structures that mandate the use of evidence for health decision making) in your country.

1. Are there legislative requirements to consider the findings of HTA in health policy decisions?
    - ○ Yes
    - ○ No

    1.1. If yes, when was this law/decree passed? Please explain in 200 words and provide the web links, if available.

    ____

2. What considerations are made in HTA analyses conducted in your country? Check all that apply.
    - ○ Safety
    - ○ Clinical efficacy/effectiveness
    - ○ Value for money or cost-effectiveness evidence
    - ○ Budget impact
    - ○ Social/ethical (e.g. equity)
    - ○ Feasibility
    - ○ Political will
    - ○ Media's interest
    - ○ Others, please specify____

3. What type of policy decisions are informed by HTA? Check all that apply.

  - ○ Introduction of drugs
  - ○ Reassessment of drugs
  - ○ Introduction of vaccines
  - ○ Reassessment of vaccines
  - ○ Introduction of devices
  - ○ Reassessment of devices
  - ○ Introduction of health screening program
  - ○ Reassessment of health screening program
  - ○ Introduction of public health interventions
  - ○ Reassessment of public health interventions
  - ○ Other, please specify____

4. Which stakeholders are involved in the HTA process? Check all those applicable.

	HTA topic nomination	HTA topic selection/prioritisation	HTA assessment	HTA appraisal	HTA result dissemination
National or Regional health authorities and policymakers					
Health care payers such as national health insurance					
Individual or Organisations of healthcare professionals					
Academics					
Patients or patient groups					
Caregiver and family member groups					

Citizen and health system user organisations not specific to any condition or disease					
Companies and associations producing health technologies					
Others					

5. What are the limitations under the HTA governance in your country? Check all that apply.

- ○ Risk of conflict of interest not well-managed
- ○ Lack of adequate funding for HTA
- ○ Lack of political support HTA process
- ○ Lack of transparency in decision making process
- ○ Risk of private sector interference
- ○ Others____

II. HTA Infrastructure

This section aims to understand the basic organisational structures and pathways that reinforce the use of HTA.

1. Are there process and method guidelines in your country? (Methods guidelines gives information about the research method to be used. Process guideline for the entire HTA process)
   - ○ Yes
   - ○ No

   1.1 If yes, please provide the weblink or name and year of publication.

   ____

2. Is there a standard definition of HTA in your country?
   - ○ Yes, (provide definition and link)
     Weblink:____
   - ○ No

3. Is there a CE threshold for your country context?
   - ○ Yes (provide value in USD)
     ____
   - ○ No

     3.1. If no, then what is the threshold used for decision making? Please provide brief detail and weblink if available.
     Weblink: ____

4. What are the limitations of the HTA infrastructure in your country?
    - ○ Lack of technical expertise to conduct HTA
    - ○ Lack of expertise in implementation of HTA institutionalisation
    - ○ Lack of standard procedures for performing HTA
    - ○ Absence of country focal point for HTA activities
    - ○ Lack of local data
    - ○ Others____

III. **Demand for HTA**

This section aims to understand who the consumers of HTA are.

1. Who are the funders of HTA?
    - ○ Public health provider
    - ○ Other government bodies
    - ○ Non-government agencies
    - ○ International agencies
    - ○ Others
2. Who are the users of HTA?
    - ○ Public health provider
    - ○ Other government bodies
    - ○ Non-government agencies
    - ○ International agencies
    - ○ Others
3. What kind of information is demanded by the users of HTA?
    - ○ Cost-effectiveness of drugs/vaccines/devices/ interventions
    - ○ Budget-impact analysis
    - ○ Equity analysis
    - ○ Feasibility analysis
    - ○ Other information:

      ____

4. What are the main barriers in translation of research into policy?
   - ○ Lack of trust in accuracy of findings
   - ○ Lack of overall political support for using HTA in policy
   - ○ Lack of transparent policy processes and procedures
   - ○ Lack of awareness about the importance and applications of HTA
   - ○ Others:____

5. Are there training/events/workshops to empower the **users** of HTA with the knowledge and uses of HTA?
   - ○ Yes
   - ○ No

   5.1 If yes, please provide name and link of such events?____
6. Is there any other limitation on the demand side?

____

IV. Supply of HTA:

This section aims to understand who the main producers of HTA are.

1. Who are the producers of HTA?
   - ○ Universities
   - ○ Institutes
   - ○ HTA focal agency within the government
   - ○ Department or teams other than HTA focal agencies
   - ○ Private sector
   - ○ Others:____

2. Please identify the top 3-5 institutions (provide names) with strong expertise for conducting a local HTA studies. Also provide links.

a.____

b.____

c.____

d.____

e.____

3. Is there a national nodal agency/department to cater to the demand from the policymakers?

- ○ Yes
- ○ No

3.1 If yes, then:

3.1.1 Is the agency autonomous?

- ○ Yes
- ○ No

3.1.2 How old is it?

- ○ 0 to 3 years
- ○ 4 to 7 years
- ○ 8 to 11 years
- ○ >11 years

3.1.3 How is it funded?

- ○ Government bodies
- ○ Private sector
- ○ International agencies
- ○ Others____

3.1.4 What are the technologies assessed by the HTA nodal agency? Check all that apply.

- ○ Drugs
- ○ Vaccines
- ○ Devices
- ○ Health screening
- ○ Public health programs
- ○ All of the above

3.1.5 What are the limitations, with regards to human resource?

- ○ Lack of experience
- ○ No in-house capacity to perform HTA

- ○ Lack of awareness about HTA
- ○ High-turnover rate
- ○ Lack of diversity in the HTA unit (For example no persons from qualitative background or having only economists etc)
- ○ Others: ____

3.1.6 What are the limitations with regards to the financing of the HTA unit?

- ○ Lack of a fixed line budget for HTA
- ○ Lack of demand of HTA
- ○ Inadequate supply of HTA
- ○ Others____

4. Does your country have formal postgraduate programs for HTA?

- ○ Yes
  - ○ <2 programs
  - ○ 2 to 4 programs
  - ○ >5 programs
- ○ No

4.1 How many Masters (top 5 universities) programs? Please name them and provide online resources if available.

a.____

b.____

c.____

d.____

e.____

4.2 How many PhD programs? Please name them and provide online resources if available.

a.____

b.____

c.____

d.____

e.____

4.3 How many informal training programs? Please name them and provide online resources if available.

a.____

b.____

c.____

d.____

e.____

4.4 Are these programs able to adequately cater to the HTA demand in your country?

- ○ Yes
- ○ No

5. Are there any other barriers in producing HTA?

____

V. Networking in HTA

This section aims to understand the global networking activities in HTA in your country.

1. What networking activities do you undertake to foster links with the international HTA community?
   - ○ Participate in international policy forums. Check all that apply.
     - ○ HTAsiaLink
     - ○ HTAi
     - ○ ISPOR
     - ○ INAHTA
     - ○ Others: ____
   - ○ Organise and host international policy forums. Please provide link

     ____

- ○ Others: ____
